# Central Adiposity, Obesity, Metabolic Syndrome, and the Risk of Thyroid Cancer in Adults Aged ≥75 Years: A Nationwide Korean Cohort Study

**DOI:** 10.3390/cancers18010049

**Published:** 2025-12-24

**Authors:** Kyung Do Han, Kwan Hoon Jo, Yunjung Cho, Hyuk-Sang Kwon, Je-Ho Han, Sung-Dae Moon, Eun Sook Kim

**Affiliations:** 1 Department of Statistics and Actuarial Science, Soongsil University, Seoul 06978, Republic of Korea; hkd917@naver.com; 2 Division of Endocrinology and Metabolism, Department of Internal Medicine, Incheon St. Mary’s Hospital, College of Medicine, The Catholic University of Korea, Incheon 21431, Republic of Korea; lovi@naver.com (K.H.J.); deity393@gmail.com (Y.C.); jehohan@gmail.com (J.-H.H.); sungdaem@gmail.com (S.-D.M.); 3 Division of Endocrinology and Metabolism, Department of Internal Medicine, Yeouido St. Mary’s Hospital, College of Medicine, The Catholic University of Korea, Seoul 07345, Republic of Korea; drkwon@catholic.ac.kr

**Keywords:** thyroid neoplasms, obesity, metabolic syndrome, abdominal obesity, aged, cohort studies

## Abstract

**Background**: The contribution of adiposity and metabolic syndrome (MetS) to thyroid cancer risk in late life, particularly among the elderly, is unclear. **Methods**: We conducted a nationwide cohort study of Korean adults aged ≥75 years who underwent standardized health screening. Exposures were body mass index (BMI), waist circumference (WC), and MetS defined by standard clinical criteria. The incidence of thyroid cancer was determined using administrative data. Fine–Gray sub-distribution hazard models estimated adjusted hazard ratios (HRs) with prespecified stratification by sex and age (75–84 vs. ≥85 years). **Results**: Among 1,164,707 participants (60.3% women), 2645 incident cases were identified. In the fully adjusted models, obesity (BMI ≥ 25 kg/m^2^) was associated with a 37% higher hazard (HR, 1.37; 95% confidence interval [CI], 1.27–1.49) and MetS with an 18% higher hazard (HR, 1.18; 95% CI, 1.09–1.28). In sex-stratified models, MetS was associated with thyroid cancer in women (HR 1.19; 95% CI, 1.08–1.31) and showed a similar direction of association in men (HR 1.16; 95% CI, 1.00–1.35), with overlapping CIs. By age, associations were evident at 75–84 years (MetS: HR, 1.18; obesity: HR, 1.36), whereas at ≥85 years, only obesity remained significant (HR, 1.90; 95% CI, 1.13–3.18). Among MetS components, high WC showed the most consistent association (HR, 1.31; 95% CI, 1.21–1.42). **Conclusions**: In adults aged ≥75 years, general obesity and, in particular, central adiposity are robustly associated with incident thyroid cancer, whereas metabolic syndrome confers a more modest and mainly age- and sex-specific additional risk.

## Simple Summary

Thyroid cancer is common in older adults, but the contribution of general and central obesity and metabolic syndrome (MetS) to thyroid cancer risk in very old populations is not well defined. Using a nationwide Korean health screening cohort of adults aged ≥75 years, we examined whether body mass index (BMI), waist circumference (WC), and MetS were associated with incident thyroid cancer, including a dedicated analysis among those aged ≥85 years. Both general obesity and central adiposity were associated with a higher risk of thyroid cancer, and MetS conferred an additional but weaker and mainly age- and sex-specific risk. Central adiposity (abdominal obesity defined by WC) showed the most consistent association across analytic models and subgroups. In contrast, the MetS association was more modest and largely confined to women aged 75–84 years, with attenuation beyond 85 years. Our findings highlight central adiposity as the dominant late-life correlate of thyroid cancer risk, while the contribution of MetS is limited to selected subgroups and becomes imprecise in the oldest-old, in whom competing mortality is substantial.

## 1. Introduction

The global incidence of thyroid cancer has risen steadily over decades, and age–period–cohort modeling projects a further 44.1% increase in cases from 2019 to 2030 [1,2]. While overdiagnosis driven by expanded imaging explains much of this rise [3], population-level analyses in some settings have reported small increases in incidence-based mortality, suggesting a modest yet clinically meaningful increase in lethal disease [4,5].

Obesity is a well-established cancer risk factor, and for thyroid cancer, a higher body mass index (BMI) has been consistently associated with a greater risk in pooled prospective cohorts and meta-analyses [6,7]. Metabolic syndrome (MetS) appears to confer an additional risk independent of BMI. In a nationwide Korean screening cohort of approximately 9.8 million adults, MetS was associated with a 15% higher risk of thyroid cancer, with a graded increase across a greater number of MetS components [8]. A recent meta-analysis of cohort studies further confirmed the positive association between obesity and thyroid cancer; however, most of the included cohorts were composed predominantly of middle-aged adults, with sparse data among individuals older than 70–75 years. As the population ages and obesity becomes more prevalent, clarifying the contribution of adiposity to thyroid cancer risk later in life has become increasingly important. Older adults often present with advanced disease and have poorer outcomes, complicating management in the context of multimorbidity and reduced physiological reserves [9,10]. However, evidence disentangling the relative contributions of adiposity and metabolic derangement in individuals aged ≥75 years is limited to a few studies and subgroup analyses [8,11]. Accordingly, we used the Korean National Health Insurance Service cohort to evaluate the associations between BMI, waist circumference (WC), MetS and incident thyroid cancer among adults aged ≥75 years, including a dedicated analysis in those aged ≥85 years.

## 2. Materials and Methods

### 2.1. Data Source Study Population

**Figure 1:**
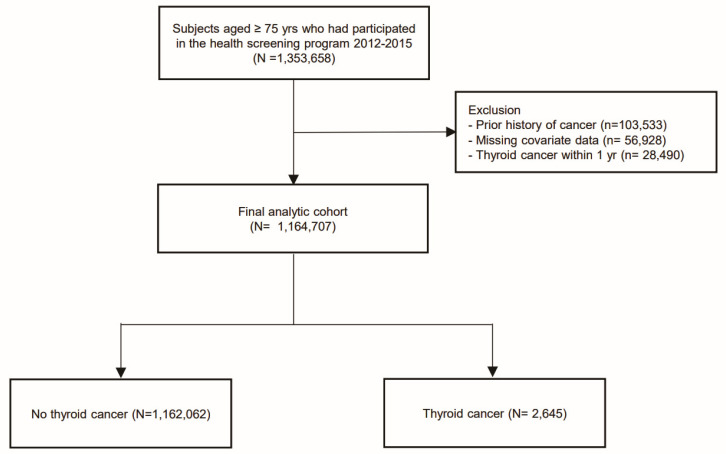
Study Flowchart.

We conducted a nationwide, population-based, retrospective cohort study using prospectively collected data from the National Health Insurance Service (NHIS) of South Korea, a single-payer program covering approximately 97% of the population that maintains comprehensive demographic, health screening, and claims records [12]. All insured individuals aged ≥40 years were invited to undergo a standardized biennial health examination. For this analysis, we identified NHIS participants aged ≥75 years who completed a health screening between 1 January 2012 and 31 December 2015 (*n* = 1,353,658) (Figure 1). We excluded participants with any recorded cancer diagnosis before the screening date, defined as at least one inpatient or outpatient claim with an ICD-10-CM code C00–C97 in combination with the cancer special co-payment code V193 during the period from 2005 (when V193 was introduced) up to the index examination (*n* = 103,533), as well as those with missing data (*n* = 56,928), yielding a baseline cohort of 1,193,197. Participants were followed from their baseline screening date (index date) until the earliest of incident thyroid cancer diagnosis, death, loss to follow-up, or 31 December 2022. Incident thyroid cancer was ascertained from the NHIS claims database using a validated claims-based algorithm: the first inpatient or outpatient claim in which the ICD-10-CM code C73 (malignant neoplasm of the thyroid gland) appeared together with the Korean cancer special co-payment reduction code V193. In the Korean National Health Insurance system, V193 denotes registration in the cancer special co-payment reduction program, which substantially reduces patient cost-sharing and requires documentation consistent with a confirmed cancer diagnosis. This definition of C73 in conjunction with V193 has been validated against the Korea Central Cancer Registry and is widely used in NHIS-based cancer epidemiologic studies [12,13]. The Institutional Review Board of The Catholic University of Korea approved the study protocol, and informed consent was waived because the data were de-identified.

### 2.2. Health Examination and Covariate Assessment

The NHIS health examination included a structured questionnaire (medical history, current medications, smoking status [never/former/current], alcohol consumption [none/light/heavy], and regular exercise), standardized anthropometrics (height, weight, and WC measured at the midpoint between the lowest rib margin and iliac crest), and laboratory tests (fasting glucose, lipid panel comprising total cholesterol, high-density lipoprotein cholesterol [HDL-C], low-density lipoprotein cholesterol [LDL-C], and triglycerides; and routine chemistry). Trained personnel performed all the measurements according to standardized protocols. BMI was calculated as weight in kilograms divided by height in meters squared, and WC was measured as previously described. Obesity was defined using the Asian-specific criteria (body mass index [BMI] ≥ 25.0 kg/m^2^, with categories < 18.5, 18.5–22.9, 23.0–24.9, 25.0–29.9, and ≥30.0 kg/m^2^) [14]. Abdominal obesity was defined as WC ≥ 90 cm in men or ≥85 cm in women [14] and further stratified into six sex-specific intervals (<80/75 cm, 80–84/75–79 cm, 85–89/80–84 cm, 90–94/85–89 cm, 95–99/90–94 cm, and ≥100/≥95 cm). Metabolic syndrome (MetS) was defined as the presence of at least three of the following modified ATP III criteria [15]: elevated triglycerides (≥150 mg/dL or use of lipid-lowering medication), low HDL-C (<40 mg/dL in men or <50 mg/dL in women or use of lipid-lowering medication), elevated blood pressure (systolic ≥ 130 mmHg or diastolic ≥ 85 mmHg or use of antihypertensive medication), elevated fasting glucose (≥100 mg/dL or use of antidiabetic medication), and abdominal obesity, as defined above. Information on smoking status, alcohol consumption, and exercise was obtained from questionnaires. Comorbidities, including diabetes mellitus (ICD-10-CM E10–E14 plus ≥ 1 antidiabetic prescription), hypertension (ICD-10-CM I10–I13 or I15 plus ≥ 1 antihypertensive prescription), dyslipidemia (ICD-10-CM E78 plus ≥ 1 lipid-lowering prescription), and chronic kidney disease (ICD-10-CM N18–N19 with eGFR < 60 mL/min/1.73 m^2^), were ascertained from claims and laboratory data during the year preceding the baseline.

### 2.3. Statistical Analysis

Baseline characteristics were summarized using means (±standard deviation [SD]) or medians (interquartile range) for continuous variables and frequencies (percentages) for categorical variables. The incidence of thyroid cancer was calculated per 1000 person-years (PY). Time-to-event analyses were performed using Fine–Gray sub-distribution hazard models, with deaths from causes other than thyroid cancer considered as competing events. We estimated sub-distribution hazard ratios (HRs) and 95% confidence intervals (CIs) for incident thyroid cancer across BMI, WC, and MetS categories using four sequential models. Model 1 was unadjusted; Model 2 adjusted for age and sex; Model 3 additionally adjusted for income, smoking status (never, former, current), alcohol consumption and physical activity; and Model 4 further adjusted for diabetes mellitus, hypertension, dyslipidemia and chronic kidney disease. BMI and WC were each analyzed in separate sets of models; we did not include BMI and WC simultaneously in the same model to limit collinearity. We assessed linear trends across BMI and sex-specific WC categories and conducted sex- and age-stratified analyses (75–84 vs. ≥85 years). Statistical analyses were performed using SAS version 9.4 (SAS Institute Inc., Cary, NC, USA). All statistical tests were two-sided, and a *p* value < 0.05 was considered significant.

## 3. Results

### 3.1. Characteristics of the Cohort

Among the 1,164,707 participants aged ≥75 years (mean ± SD 78.7 ± 3.6; 60.3% women), 2645 incident thyroid cancers accrued over 8,038,565 person-years (PY) (incidence rate, 0.33 per 1000 PY; median follow-up, 6.9 years). Compared with participants who remained cancer-free (*n* = 1,162,062), those who developed thyroid cancer (*n* = 2645) were slightly younger (77.7 ± 2.7 vs. 78.7 ± 3.6 years; *p* < 0.001), less often male (32.3% vs. 39.8%; *p* < 0.001), and had higher BMI (24.3 ± 3.1 vs. 23.5 ± 3.3 kg/m^2^; *p* < 0.001) and WC (84.3 ± 8.5 vs. 82.9 ± 8.9 cm; *p* < 0.001). Non-smoking (81.6% vs. 77.9%; *p* < 0.001) and non-drinking (85.4% vs. 83.3%; *p* = 0.009) were more common, while hypertension (75.1% vs. 72.2%; *p* = 0.001) and dyslipidemia (41.2% vs. 38.7%; *p* = 0.008) were more prevalent in the incident cancer group (Table 1).

### 3.2. Metabolic Syndrome and Obesity

In multivariable Fine–Gray competing-risk models (Model 4; Table 2), metabolic syndrome (MetS) was associated with an 18% higher risk of thyroid cancer (HR, 1.18; 95% CI, 1.09–1.28; *p* < 0.001), and obesity (BMI ≥ 25 kg/m^2^) was associated with a 37% higher risk (HR, 1.37; 95% CI, 1.27–1.49; *p* < 0.001). In sex-stratified analyses, MetS was significant in women (HR, 1.19; 95% CI, 1.08–1.31; *p* < 0.001) but borderline in men (HR, 1.16; 95% CI, 1.00–1.35; *p* = 0.049), whereas obesity was significant in both women (HR, 1.36; 95% CI, 1.24–1.50; *p* < 0.001) and men (HR, 1.40; 95% CI, 1.21–1.62; *p* < 0.001). In age-stratified analyses, both MetS and obesity were associated with a higher risk of thyroid cancer in the 75–84-year age group. Among participants aged ≥85 years, MetS was not significantly associated with thyroid cancer (HR 1.22; 95% CI 0.73–2.03), and the wide confidence interval is compatible with both no effect and up to approximately a two-fold higher hazard. In contrast, obesity remained significantly associated with incident thyroid cancer (HR 1.90; 95% CI 1.13–3.18), although this estimate is based on a limited number of events in subjects ≥ 85 years and should be interpreted with caution.

### 3.3. Combined Effects of MetS and Obesity

The participants were further categorized according to their joint MetS/obesity status (Table 3). Compared with neither condition, the risk was higher for obesity alone (HR, 1.49), MetS alone (HR, 1.21), and the coexistence of both (HR, 1.42), with no evidence of a multiplicative interaction between obesity and MetS.

### 3.4. Dose–Response Relationships

**Figure 2:**
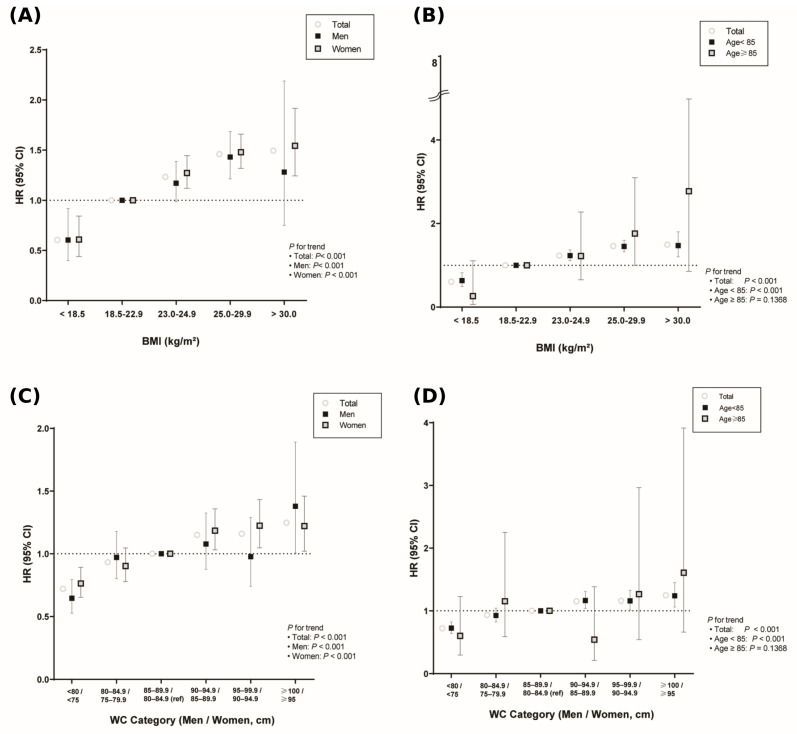
Associations of BMI and Waist Circumference with Incident Thyroid Cancer, Stratified by Sex and by Age Group. Hazard ratios (HRs) and 95% confidence intervals (CIs) for incident thyroid cancer according to (**A**,**B**) five BMI categories and (**C**,**D**) six sex-specific waist-circumference (WC) categories, estimated from Model 4 (adjusted for age, sex [unless stratified], income, smoking, alcohol, physical activity, diabetes mellitus, hypertension, dyslipidemia, and chronic kidney disease). The horizontal dashed line at HR = 1 indicates the reference category in each panel. Panel (**A**) (**top left**): Total cohort vs. men vs. women. Panel (**B**) (**top right**): Total cohort vs. age < 85 years vs. age ≥ 85 years. Panel (**C**) (**bottom left**): Total cohort vs. men vs. women according to sex-specific WC. Panel (**D**) (**bottom right**): Total cohort vs. age < 85 years vs. age ≥ 85 years, according to sex-specific WC.

There were monotonic increases in thyroid cancer hazard across ascending BMI and sex-specific WC categories in the overall cohort, in men and women, and in those aged 75–84 years (*p* for trend < 0.001 for all). No clear trend was observed among participants aged ≥85 years (*p* = 0.137) (Figure 2).

### 3.5. Individual MetS Components

In the total population, high WC (Model 4 HR, 1.31; 95% CI, 1.21–1.42; *p* < 0.001), low HDL-C (HR, 1.10; 95% CI, 1.02–1.19; *p* = 0.019), and high blood pressure (HR, 1.09; 95% CI, 1.01–1.18; *p* = 0.048) were independently associated with incident thyroid cancer, whereas high triglyceride and elevated fasting glucose levels were not (both *p* > 0.05) (Appendix A Table A1). Sex- and age-specific component analyses (Appendix A Table A2) showed that high WC remained the strongest predictor in both men and women and in participants aged 75–84 years, with attenuation in the oldest subgroup (≥85 years).

Collectively, MetS and general obesity, particularly central adiposity indexed by high WC, were independently associated with thyroid cancer risk in older adults, with the strongest and most consistent associations observed in women and individuals aged 75–84 years.

## 4. Discussion

### 4.1. Summary of Principal Findings

In this nationwide cohort of adults aged ≥75 years, both general adiposity (BMI) and central adiposity (WC) were consistently associated with a higher risk of incident thyroid cancer, whereas MetS showed a weaker sex-dependent association with thyroid cancer risk. After multivariable adjustment, obesity (BMI ≥ 25 kg/m^2^) was associated with a 37% higher hazard of thyroid cancer, and MetS with an 18% higher hazard. These associations were evident in adults aged 75–84 years; among those aged ≥85 years, only obesity remained significantly associated with thyroid cancer risk. Among the individual MetS components, abdominal adiposity (high WC) exhibited the most robust and consistent association with incident thyroid cancer in this study. In the joint analyses, obesity alone, MetS alone, and their coexistence each conferred a higher risk than the absence of both, with no evidence of multiplicative interaction. To limit collinearity, our primary models treated BMI and WC in separate model sets rather than including both in the same model. In an exploratory fully adjusted model that included both BMI and WC, the variance inflation factors for BMI and WC were approximately 2.5 and all covariates had VIFs < 3, indicating only modest multicollinearity and supporting WC as a robust multivariable correlate of late-life thyroid cancer risk.

### 4.2. Comparison with Prior Literature

Our findings align with those of pooled prospective cohorts and meta-analyses showing a dose-related positive association between BMI and thyroid cancer risk [6,16]. In a large Asian cohort from the Korean NHIS (*n* = 4,658,473; age 40–70 years), thyroid cancer risk increased progressively with the number of MetS components [8], whereas a European consortium analysis (Me-Can; *n* = 578,700; mean baseline age 44 years) did not show a consistent association with composite MetS indices [17]. Against this heterogeneous background, our study characterized the late-life pattern: BMI-related risk persists at ≥75 years, MetS appears informative mainly among older women, and adiposity predominates beyond the age of 85 years.

### 4.3. Biological Plausibility

Obesity and its metabolic sequelae create a pro-carcinogenic environment that may promote the initiation and progression of thyroid cancer. Hyperinsulinemia with downstream IGF-1/IGF-1R signaling activates the PI3K–AKT–mTOR and MAPK pathways, supporting the survival and clonal expansion of mutated thyrocytes in the context of chronic low-grade inflammation [18]. Simultaneously, adipokine imbalance (higher leptin and lower adiponectin levels) augments proliferative, anti-apoptotic, and pro-angiogenic signaling [19,20]. Visceral adiposity is particularly metabolically active, integrating insulin resistance, oxidative stress and endothelial dysfunction [21]. In addition, obesity is often accompanied by modest elevations in TSH; higher TSH levels have been linked to malignancy among thyroid nodules and to adverse tumor features in differentiated thyroid cancer, suggesting a TSH-mediated proliferative axis that could potentiate adiposity-related carcinogenic pathways [22]. In our study, however, TSH and other thyroid function tests were not available, so this mechanism cannot be directly evaluated and should be regarded as a plausible but unproven explanation for the observed associations.

### 4.4. Age-Specific Findings

Beyond 85 years, only obesity retained a clear association with incident thyroid cancer, whereas MetS did not. The dose–response for BMI and WC that was evident at ages 75–84 diminished thereafter, likely reflecting: (i) survivor and selection biases that enrich the oldest-old with metabolically “hardier” individuals; (ii) lower diagnostic intensity and under-ascertainment of indolent disease; (iii) age-related changes in body composition (sarcopenia, fat redistribution, and height loss) that increase misclassification of adiposity by BMI or WC; (iv) stronger competing risks (non-cancer mortality) that shorten the time window to detect cancers with long latency; and (v) treatment-related and definitional issues that blunt MetS signals in late life (medication use normalizing MetS components, threshold misfit, and weight loss from comorbid illness) [23,24,25,26]. Together, these factors can obscure weaker composite indices, such as MetS, while allowing for a more robust effect of obesity.

### 4.5. Sex Differences

MetS was associated with a higher risk in women and showed a similar, albeit less precise, association in men, whereas obesity was associated with risk in both sexes. The CIs for men and women overlapped, and although post hoc interaction tests did not provide strong statistical evidence of a sex × MetS interaction, these analyses were not prespecified. Therefore, the sex differences should be interpreted as suggestive rather than definitive. The upstream mechanisms of insulin/IGF-1 signaling, low-grade inflammation, adipokine imbalance, and TSH-related stimulation are likely shared across sexes; however, effect magnitudes may diverge in postmenopausal women owing to (i) greater reliance on adipose aromatase–derived estrogen, (ii) lower sex hormone–binding globulin (SHBG) in MetS increases bioavailable estradiol, and (iii) estrogen receptor (ER) and G-protein-coupled estrogen receptor (GPER) signaling within thyroid tissue [27,28,29]. These factors can amplify the same downstream cascades, yielding a stronger MetS signal in women, even as obesity elevates the risk in both sexes.

### 4.6. Clinical Implications

In late life, excess adiposity remains a meaningful risk factor for incident thyroid cancer, indicating that, where feasible and safe, weight management and reduction in central adiposity should continue to be discussed as preventive targets in adults aged ≥75 years. Because MetS is associated with risk, particularly among women aged 75–84 years, it is reasonable to prioritize the optimization of blood pressure and lipid levels, even when the BMI alone does not appear elevated.

These findings do not support population screening; rather, they argue for risk-attuned clinical vigilance—for example, careful evaluation of nodular thyroid disease in older adults with obesity or MetS—while adhering to guidance that cautions against overdiagnosis. Among the oldest-old (≥85 years), in which obesity continues to signal risk, but competing risks are substantial, evaluation and management should be individualized through shared decision-making that explicitly considers frailty, comorbidities, and patient goals.

### 4.7. Strengths and Limitations

This study has several strengths. It was conducted in a large, nationwide cohort of adults aged ≥75 years, with near-complete coverage of the Korean population and linkage to comprehensive health screening and claims data. The size of the cohort and the long follow-up allowed us to examine age- and sex-specific patterns, including a dedicated subgroup of adults aged ≥85 years, who are often under-represented in cancer epidemiology. Detailed information on anthropometry, metabolic comorbidities and lifestyle factors enabled multivariable adjustment for key confounders and evaluation of central adiposity as a late-life correlate of thyroid cancer risk. However, several limitations should be acknowledged. First, we relied on a claims-based algorithm (C73 in conjunction with V193) to ascertain incident thyroid cancer. Although this definition has been validated against the national cancer registry and is widely used in Korean NHIS-based research, detailed information on tumor stage, histology and pathology was not available, and some degree of misclassification cannot be excluded. Second, we were unable to systematically identify autoimmune thyroid disorders such as Graves’ disease or chronic autoimmune thyroiditis because thyroid function tests, thyroid auto-antibodies, and detailed thyroid imaging/pathology data were not available in this NHIS screening dataset. Some of these conditions may increase the risk of thyroid cancer and could therefore confound or modify the observed associations between adiposity, metabolic syndrome, and incident thyroid cancer. Some residual confounding by unmeasured autoimmune thyroid disease cannot be excluded. Third, we could not reliably identify rare hereditary cancer predisposition syndromes (e.g., familial medullary thyroid carcinoma or other germline mutations associated with endocrine neoplasia) in this administrative dataset. While such conditions account for a small fraction of thyroid cancers in the general population, we cannot exclude a modest contribution of unmeasured genetic susceptibility to the observed associations. Fourth, although smoking is an important behavioral risk factor and potential effect modifier, current smoking was relatively uncommon in this very elderly cohort, particularly among women and those aged ≥85 years, limiting the power to detect modest interactions. In exploratory analyses, including multiplicative interaction terms between MetS or obesity and smoking status, the *p* values for interaction were all >0.3 across the total cohort and sex- and age-stratified models, except for an unstable estimate in the ≥85-year ex-/current smoking subgroup with extremely sparse events. These findings do not provide strong evidence that smoking materially modifies the associations of MetS or obesity with thyroid cancer risk, but they cannot exclude small interaction effects. Relatedly, we lacked TSH and other thyroid function indices, precluding the direct assessment of thyroid functional status as a mediator or confounder of the adiposity–thyroid cancer association. In addition, we could not fully account for potential detection bias: individuals with obesity or multiple comorbidities may undergo more intensive thyroid evaluation (for example, more frequent ultrasound examinations), which could increase the likelihood of detecting indolent thyroid cancers and partially influence the observed associations. Finally, we lacked individual-level data on several established or suspected determinants of thyroid cancer, including dietary iodine intake, prior therapeutic or environmental radiation exposure to the neck, and family history or genetic susceptibility. We therefore cannot exclude residual confounding by these unmeasured factors, although such exposures are unlikely to fully account for the consistent associations observed for central adiposity across multiple subgroups. In particular, the MetS estimate in adults aged ≥85 years (HR 1.22; 95% CI 0.73–2.03) should be interpreted cautiously, as the wide CI is compatible with both no association and a clinically relevant increase in risk, and the obesity association (HR 1.90; 95% CI 1.13–3.18) may be influenced by competing risks in this age group.

## 5. Conclusions

In a nationwide cohort of community-dwelling adults aged ≥75 years, general obesity and, in particular, central adiposity were robust risk factors for incident thyroid cancer across sexes, whereas MetS conferred a more modest additional risk, primarily in women aged 75–84 years. These data support adiposity, especially central adiposity, as a clinically relevant target for risk reduction and reinforce the importance of metabolic optimization in older women within a framework that avoids indiscriminate screening and prioritizes individualized care.

## Figures and Tables

**Table 1:** Baseline Characteristics of Participants by Thyroid Cancer Status.

	No Thyroid Cancer	Thyroid Cancer	*p*
	(*n* = 1,162,062)	(*n* = 2645)	
Age group			<0.001
75–84 years	1,078,258 (92.8%)	2576 (97.4%)	
≥85 years	83,804 (7.2%)	69 (2.6%)	
Sex, male	462,093 (39.8%)	853 (32.3%)	<0.001
BMI			<0.001
<18.5 kg/m^2^	63,002 (5.4%)	63 (2.4%)	
18.5–22.9 kg/m^2^	445,969 (38.4%)	819 (31.0%)	
23.0–24.9 kg/m^2^	284,414 (24.5%)	676 (25.6%)	
25.0–29.9 kg/m^2^	333,112 (28.7%)	975 (36.9%)	
≥30.0 kg/m^2^	35,565 (3.1%)	112 (4.2%)	
Waist Circumference			<0.001
<80/75 cm	268,619 (23.1%)	424 (16.0%)	
80/75–84/79 cm	237,476 (20.4%)	508 (19.2%)	
85/80–89/84 cm	268,067 (23.1%)	630 (23.8%)	
90/85–94/89 cm	200,358 (17.2%)	546 (20.6%)	
95/90–99/94 cm	114,357 (9.8%)	318 (12.0%)	
≥100/95 cm	73,185 (6.3%)	219 (8.3%)	
Smoking status			<0.001
Non-smoker	904,894 (77.9%)	2158 (81.6%)	
Ex-smoker	171,878 (14.8%)	318 (12.0%)	
Current smoker	85,290 (7.3%)	169 (6.4%)	
Drinking status			0.009
Non-drinker	967,623 (83.3%)	2260 (85.4%)	
Mild drinker	163,707 (14.1%)	329 (12.4%)	
Heavy drinker	30,732 (2.6%)	56 (2.1%)	
Regular exercise	168,984 (14.5%)	422 (16.0%)	0.04
Diabetes mellitus	276,479 (23.8%)	602 (22.8%)	0.213
Hypertension	838,385 (72.2%)	1986 (75.1%)	0.001
Dyslipidemia	449,518 (38.7%)	1090 (41.2%)	0.008
CKD	324,167 (27.9%)	643 (24.3%)	<0.001
Age (years)	78.7 ± 3.6	77.7 ± 2.7	<0.001
BMI (kg/m^2^)	23.5 ± 3.3	24.3 ± 3.1	<0.001
WC (cm)	82.9 ± 8.9	84.3 ± 8.5	<0.001
Systolic BP (mmHg)	130.8 ± 16.0	131.7 ± 15.9	0.005
Diastolic BP (mmHg)	77.1 ± 10.0	77.8 ± 10.1	<0.001
HDL-C (mg/dL)	52.2 ± 14.1	51.6 ± 13.0	0.041
LDL-C (mg/dL)	111.4 ± 35.5	110.8 ± 35.0	0.379
Fasting glucose (mg/dL)	104.9 ± 27.2	104.1 ± 24.7	0.122
Total cholesterol (mg/dL)	189.8 ± 39.3	188.7 ± 37.9	0.145
Triglycerides (mg/dL) *	116.7 (116.6–116.8)	117.0 (115.0–119.1)	<0.001

Values are presented as *n* (%) for categorical variables and mean ± standard deviation (SD) for continuous variables. * TG values are expressed as geometric means with 95% confidence intervals (CIs). Abbreviations: BP, blood pressure; CKD, chronic kidney disease; BMI, body mass index; WC, waist circumference; HDL-C, high-density lipoprotein cholesterol; LDL-C, low-density lipoprotein cholesterol.

**Table 2:** Association of Metabolic Syndrome (MetS) and Obesity with Incident Thyroid Cancer.

Subgroup	Variable	*n*	Events	IR per 1000 PY	Model 1 HR (95% CI)	Model 2 HR (95% CI)	Model 3 HR (95% CI)	Model 4 HR (95% CI)
Total	MetS−	757,931	1598	0.31	1.00 (ref.)	1.00 (ref.)	1.00 (ref.)	1.00 (ref.)
	MetS+	406,776	1047	0.37	1.22 (1.13, 1.32)	1.17 (1.08, 1.26)	1.17 (1.08, 1.26)	1.18 (1.09, 1.28)
	*p*				<0.001	<0.001	<0.001	<0.001
	BMI < 25.0 kg/m^2^	794,943	1558	0.29	1.00 (ref.)	1.00 (ref.)	1.00 (ref.)	1.00 (ref.)
	BMI ≥ 25.0 kg/m^2^	369,764	1087	0.41	1.51 (1.40, 1.63)	1.38 (1.27, 1.49)	1.38 (1.27, 1.49)	1.37 (1.27, 1.49)
	*p*				<0.001	<0.001	<0.001	<0.001
Men	MetS−	337,661	599	0.27	1.00 (ref.)	1.00 (ref.)	1.00 (ref.)	1.00 (ref.)
	MetS+	125,285	254	0.31	1.15 (0.99, 1.33)	1.13 (0.97, 1.31)	1.13 (0.98, 1.31)	1.16 (1.00, 1.35)
	*p*				0.068	0.106	0.101	0.049
	BMI < 25.0 kg/m^2^	340,404	566	0.26	1.00 (ref.)	1.00 (ref.)	1.00 (ref.)	1.00 (ref.)
	BMI ≥ 25.0 kg/m^2^	122,542	287	0.34	1.42 (1.23, 1.63)	1.37 (1.19, 1.58)	1.37 (1.19, 1.58)	1.40 (1.21, 1.62)
	*p*				<0.001	<0.001	<0.001	<0.001
Women	MetS−	420,270	999	0.33	1.00 (ref.)	1.00 (ref.)	1.00 (ref.)	1.00 (ref.)
	MetS+	281,491	793	0.4	1.19 (1.08, 1.30)	1.18 (1.08, 1.30)	1.19 (1.08, 1.30)	1.19 (1.08, 1.31)
	*p*				<0.001	<0.001	<0.001	0.001
	BMI < 25.0 kg/m^2^	454,539	992	0.31	1.00 (ref.)	1.00 (ref.)	1.00 (ref.)	1.00 (ref.)
	BMI ≥ 25.0 kg/m^2^	247,222	800	0.44	1.49 (1.36, 1.64)	1.38 (1.26, 1.51)	1.38 (1.26, 1.51)	1.36 (1.24, 1.50)
	*p*				<0.001	<0.001	<0.001	<0.001
Age 75–84	MetS−	701,522	1555	0.32	1.00 (ref.)	1.00 (ref.)	1.00 (ref.)	1.00 (ref.)
	MetS+	379,312	1021	0.38	1.22 (1.12, 1.32)	1.16 (1.07, 1.26)	1.17 (1.08, 1.26)	1.18 (1.08, 1.28)
	*p*				<0.001	<0.001	<0.001	<0.001
	BMI < 25.0 kg/m^2^	727,495	1512	0.3	1.00 (ref.)	1.00 (ref.)	1.00 (ref.)	1.00 (ref.)
	BMI ≥ 25.0 kg/m^2^	353,339	1064	0.42	1.46 (1.35, 1.58)	1.37 (1.26, 1.48)	1.37 (1.26, 1.48)	1.36 (1.26, 1.48)
	*p*				<0.001	<0.001	<0.001	<0.001
Age ≥ 85	MetS−	56,409	43	0.15	1.00 (ref.)	1.00 (ref.)	1.00 (ref.)	1.00 (ref.)
	MetS+	27,464	26	0.18	1.24 (0.76, 2.02)	1.24 (0.75, 2.03)	1.24 (0.75, 2.04)	1.22 (0.73, 2.03)
	*p*				0.380	0.406	0.402	0.454
	BMI < 25.0 kg/m^2^	67,448	46	0.14	1.00 (ref.)	1.00 (ref.)	1.00 (ref.)	1.00 (ref.)
	BMI ≥ 25.0 kg/m^2^	16,425	23	0.24	2.06 (1.25, 3.39)	1.99 (1.22, 3.26)	1.95 (1.19, 3.20)	1.90 (1.13, 3.18)
	*p*				0.005	0.006	0.008	0.015

PY, person-years; HR, hazard ratio; CI, confidence interval; MetS, metabolic syndrome. Model 1 was non-adjusted. Model 2 was adjusted for age and sex (where applicable). Model 3 was additionally adjusted for income, smoking status, alcohol consumption, and physical activity. Model 4 was further adjusted for comorbidities (diabetes mellitus, hypertension, dyslipidemia, and CKD.

**Table 3:** Hazard Ratios for Incident Thyroid Cancer by Combined Metabolic Syndrome and Obesity Status, Overall and by Sex and Age Subgroup.

	MetS and Obesity	*n*	Events	PY	IR per 1000 PY	Model 1 HR (95% CI)	Model 2 HR (95% CI)	Model 3 HR (95% CI)	Model 4 HR (95% CI)
Total	(−) & (−)	597,915	1111	4,054,226.80	0.27	1.00 (ref.)	1.00 (ref.)	1.00 (ref.)	1.00 (ref.)
	(−) & (+)	160,016	487	1,159,306.59	0.42	1.65 (1.48–1.83)	1.50 (1.35–1.67)	1.50 (1.35–1.67)	1.49 (1.34–1.66)
	(+) & (−)	197,028	447	1,330,252.59	0.34	1.22 (1.10–1.36)	1.19 (1.06–1.32)	1.19 (1.07–1.33)	1.21 (1.08–1.35)
	(+) & (+)	209,748	600	1,494,779.76	0.40	1.55 (1.40–1.71)	1.40 (1.27–1.55)	1.40 (1.27–1.55)	1.42 (1.28–1.58)
	*p* for trend					<0.0001	<0.0001	<0.0001	<0.0001
Male	(−) & (−)	278,328	456	1,803,270.40	0.25	1.00 (ref.)	1.00 (ref.)	1.00 (ref.)	1.00 (ref.)
	(−) & (+)	59,333	143	411,302.23	0.35	1.48 (1.23–1.79)	1.43 (1.18–1.72)	1.43 (1.19–1.73)	1.45 (1.20–1.75)
	(+) & (−)	62,076	110	393,423.42	0.28	1.08 (0.88–1.34)	1.08 (0.87–1.33)	1.08 (0.88–1.33)	1.12 (0.90–1.38)
	(+) & (+)	63,209	144	427,966.35	0.34	1.40 (1.16–1.69)	1.35 (1.12–1.63)	1.36 (1.12–1.64)	1.41 (1.17–1.72)
	*p* for trend					<0.0001	0.0002	0.0002	<0.0001
Female	(−) & (−)	319,587	655	2,250,956.41	0.29	1.00 (ref.)	1.00 (ref.)	1.00 (ref.)	1.00 (ref.)
	(−) & (+)	100,683	344	748,004.36	0.46	1.68 (1.47–1.91)	1.54 (1.35–1.76)	1.54 (1.35–1.75)	1.52 (1.33–1.73)
	(+) & (−)	134,952	337	936,829.18	0.36	1.22 (1.07–1.39)	1.24 (1.09–1.42)	1.25 (1.09–1.42)	1.26 (1.10–1.43)
	(+) & (+)	146,539	456	1,066,813.41	0.43	1.53 (1.35–1.72)	1.43 (1.27–1.62)	1.44 (1.27–1.62)	1.44 (1.27–1.63)
	*p* for trend					<0.0001	<0.0001	<0.0001	<0.0001
Age < 85	(−) & (−)	548,177	1079	3,814,253.73	0.28	1.00 (ref.)	1.00 (ref.)	1.00 (ref.)	1.00 (ref.)
	(−) & (+)	153,345	476	1,120,811.61	0.42	1.59 (1.42–1.77)	1.48 (1.33–1.65)	1.48 (1.33–1.65)	1.47 (1.32–1.64)
	(+) & (−)	179,318	433	1,241,706.65	0.35	1.23 (1.10–1.37)	1.19 (1.06–1.33)	1.19 (1.06–1.33)	1.21 (1.08–1.35)
	(+) & (+)	199,994	588	1,438,781.71	0.41	1.50 (1.36–1.66)	1.39 (1.26–1.54)	1.40 (1.26–1.54)	1.42 (1.28–1.57)
	*p*					<0.0001	<0.0001	<0.0001	<0.0001
Age ≥ 85	(−) & (−)	49,738	32	239,973.07	0.13	1.00 (ref.)	1.00 (ref.)	1.00 (ref.)	1.00 (ref.)
	(−) & (+)	6671	11	38,494.99	0.29	2.57 (1.30–5.09)	2.49 (1.26–4.93)	2.44 (1.23–4.85)	2.34 (1.16–4.69)
	(+) & (−)	17,710	14	88,545.95	0.16	1.23 (0.66–2.31)	1.24 (0.66–2.32)	1.25 (0.67–2.33)	1.24 (0.66–2.33)
	(+) & (+)	9754	12	55,998.05	0.21	1.92 (0.99–3.72)	1.86 (0.96–3.62)	1.83 (0.94–3.56)	1.80 (0.90–3.59)
	*p* for trend					0.0289	0.0357	0.0453	0.0749

## Data Availability

The data used in this study were obtained from the National Health Insurance Service (NHIS) of South Korea. Restrictions apply to the availability of these data, which were used under license for the current study and are not publicly available due to privacy and ethical considerations.

## Abbreviations

The following abbreviations are used in this manuscript:

BMI	Body mass index
MetS	Metabolic syndrome
WC	Waist circumference
NHIS	National Health Insurance Service
HDL-C	High-density lipoprotein cholesterol
LDL-C	Low-density lipoprotein cholesterol
SD	Standard deviation
PY	Person-years
HRs	Hazard ratios
CIs	Confidence intervals
SHBG	Sex hormone-binding globulin
ER	Estrogen receptor
GPER	G-protein-coupled estrogen receptor

## Appendix A

Appendix A Table A1 and Table A2 present the incidence and hazard ratios of thyroid cancer according to individual metabolic syndrome components in the total population and by sex and age group.

**Table A1:** Incidence of Thyroid Cancer According to Individual Metabolic Syndrome Components in the Total Population.

Component	Category	*n*	Events	PY	IR per 1000 PY	Model 1 HR (95% CI)	Model 2 HR (95% CI)	Model 3 HR (95% CI)	Model 4 HR (95% CI)
High WC	No	775,724	1562	5,301,181.69	0.29	1.00 (ref.)	1.00 (ref.)	1.00 (ref.)	1.00 (ref.)
	Yes	388,983	1083	2,737,384.05	0.40	1.39 (1.29–1.50)	1.31 (1.21–1.42)	1.31 (1.21–1.42)	1.31 (1.21–1.42)
	*p*					<0.001	<0.001	<0.001	<0.001
High TG	No	827,441	1860	5,683,789.07	0.33	1.00 (ref.)	1.00 (ref.)	1.00 (ref.)	1.00 (ref.)
	Yes	337,266	785	2,354,776.68	0.33	1.03 (0.95–1.12)	1.00 (0.92–1.08)	1.00 (0.92–1.09)	1.00 (0.92–1.09)
	*p*					0.438	0.908	0.994	0.988
High BP	No	482,140	1019	3,289,713.31	0.31	1.00 (ref.)	1.00 (ref.)	1.00 (ref.)	1.00 (ref.)
	Yes	682,567	1626	4,748,852.44	0.34	1.13 (1.04–1.22)	1.12 (1.03–1.21)	1.12 (1.04–1.21)	1.09 (1.01–1.18)
	*p*					0.003	0.005	0.004	0.049
High Glucose	No	611,797	1411	4,269,520.39	0.33	1.00 (ref.)	1.00 (ref.)	1.00 (ref.)	1.00 (ref.)
	Yes	552,910	1234	3,769,045.36	0.33	0.98 (0.91–1.05)	0.98 (0.91–1.05)	0.98 (0.91–1.05)	0.99 (0.91–1.08)
	*p*					0.454	0.540	0.546	0.844
Low HDL	No	730,917	1575	5,077,612.75	0.31	1.00 (ref.)	1.00 (ref.)	1.00 (ref.)	1.00 (ref.)
	Yes	433,790	1070	2,960,953.00	0.36	1.15 (1.06–1.24)	1.09 (1.01–1.18)	1.09 (1.01–1.19)	1.10 (1.02–1.19)
	*p*					0.001	0.030	0.028	0.019

**Table A2:** Incidence of Thyroid Cancer According to Individual Metabolic Syndrome Components by Sex and Age Group.

	Component	Category	*n*	Events	PY	IR per 1000 PY	Model 1 HR (95% CI)	Model 2 HR (95% CI)	Model 3 HR (95% CI)	Model 4 HR (95% CI)
Men	High WC	No	335,206	579	2,182,318.23	0.27	1.00 (ref.)	1.00 (ref.)	1.00 (ref.)	1.00 (ref.)
		Yes	127,740	274	853,644.16	0.32	1.25 (1.08–1.44)	1.23 (1.06–1.41)	1.23 (1.06–1.42)	1.25 (1.08–1.45)
		*p*					0.003	0.006	0.006	0.003
	High TG	No	349,280	646	2,285,208.46	0.28	1.00 (ref.)	1.00 (ref.)	1.00 (ref.)	1.00 (ref.)
		Yes	113,666	207	750,753.92	0.28	0.99 (0.84–1.15)	0.96 (0.82–1.13)	0.97 (0.83–1.13)	0.99 (0.84–1.15)
		*p*					0.845	0.636	0.678	0.852
	High BP	No	200,219	358	1,303,388.91	0.27	1.00 (ref.)	1.00 (ref.)	1.00 (ref.)	1.00 (ref.)
		Yes	262,727	495	1,732,573.47	0.29	1.05 (0.92–1.21)	1.05 (0.92–1.21)	1.06 (0.92–1.21)	1.01 (0.87–1.16)
		*p*					0.465	0.451	0.430	0.917
	High Glucose	No	237,797	446	1,574,059.78	0.28	1.00 (ref.)	1.00 (ref.)	1.00 (ref.)	1.00 (ref.)
		Yes	225,149	407	1,461,902.61	0.28	0.97 (0.85–1.11)	0.96 (0.84–1.10)	0.96 (0.84–1.10)	1.01 (0.87–1.16)
		*p*					0.564	0.561	0.936	0.564
	Low HDL	No	354,030	646	2,345,839.70	0.28	1.00 (ref.)	1.00 (ref.)	1.00 (ref.)	1.00 (ref.)
		Yes	108,916	207	690,122.69	0.30	1.05 (0.89–1.22)	1.05 (0.90–1.23)	1.05 (0.89–1.23)	1.06 (0.90–1.24)
		*p*					0.584	0.550	0.564	0.511
Women	High WC	No	440,518	983	3,118,863.47	0.32	1.00 (ref.)	1.00 (ref.)	1.00 (ref.)	1.00 (ref.)
		Yes	261,243	809	1,883,739.89	0.43	1.40 (1.27–1.53)	1.35 (1.23–1.48)	1.35 (1.23–1.48)	1.34 (1.22–1.47)
		*p*					<0.001	<0.001	<0.001	<0.001
	High TG	No	478,161	1214	3,398,580.60	0.36	1.00 (ref.)	1.00 (ref.)	1.00 (ref.)	1.00 (ref.)
		Yes	223,600	578	1,604,022.76	0.36	1.02 (0.92–1.12)	1.01 (0.92–1.12)	1.02 (0.92–1.12)	1.01 (0.91–1.12)
		*p*					0.764	0.814	0.735	0.849
	High BP	No	281,921	661	1,986,324.40	0.33	1.00 (ref.)	1.00 (ref.)	1.00 (ref.)	1.00 (ref.)
		Yes	419,840	1131	3,016,278.96	0.37	1.15 (1.04–1.26)	1.15 (1.05–1.27)	1.15 (1.05–1.27)	1.12 (1.02–1.24)
		*p*					0.005	0.004	0.003	0.023
	High Glucose	No	374,000	965	2,695,460.61	0.36	1.00 (ref.)	1.00 (ref.)	1.00 (ref.)	1.00 (ref.)
		Yes	327,761	827	2,307,142.75	0.36	0.99 (0.90–1.08)	0.99 (0.90–1.08)	0.99 (0.90–1.08)	0.98 (0.89–1.09)
		*p*					0.764	0.780	0.757	0.764
	Low HDL	No	376,887	929	2,731,773.05	0.34	1.00 (ref.)	1.00 (ref.)	1.00 (ref.)	1.00 (ref.)
		Yes	324,874	863	2,270,830.31	0.38	1.08 (0.98–1.18)	1.11 (1.01–1.22)	1.11 (1.02–1.22)	1.13 (1.03–1.24)
		*p*					0.111	0.028	0.024	0.014
Age < 85	High WC	No	714,703	1514	5,003,212.39	0.3	1.00 (ref.)	1.00 (ref.)	1.00 (ref.)	1.00 (ref.)
		Yes	366,131	1,062	2,612,341.30	0.41	1.38 (1.27–1.49)	1.31 (1.22–1.42)	1.32 (1.22–1.42)	1.32 (1.22–1.43)
		*p*					<0.001	<0.001	<0.001	<0.001
	High TG	No	765,431	1,806	5,376,589.48	0.34	1.00 (ref.)	1.00 (ref.)	1.00 (ref.)	1.00 (ref.)
		Yes	315,403	770	2,238,964.21	0.34	1.03 (0.95–1.12)	1.00 (0.92–1.09)	1.01 (0.93–1.10)	1.01 (0.93–1.10)
		*p*					0.455	0.976	0.882	0.868
	High BP	No	446,288	995	3,118,852.19	0.32	1.00 (ref.)	1.00 (ref.)	1.00 (ref.)	1.00 (ref.)
		Yes	634,546	1581	4,496,701.50	0.35	1.12 (1.03–1.21)	1.11 (1.03–1.21)	1.12 (1.03–1.21)	1.08 (1.00–1.18)
		*p*					0.007	0.009	0.007	0.063
	High Glucose	No	567,289	1376	4,045,532.82	0.34	1.00 (ref.)	1.00 (ref.)	1.00 (ref.)	1.00 (ref.)
		Yes	513,545	1200	3,570,020.87	0.34	0.97 (0.90–1.05)	0.97 (0.90–1.05)	0.97 (0.90–1.05)	0.99 (0.91–1.08)
		*p*					0.397	0.502	0.512	0.791
	Low HDL	No	683,986	1534	4,831,468.17	0.32	1.00 (ref.)	1.00 (ref.)	1.00 (ref.)	1.00 (ref.)
		Yes	396,848	1042	2,784,085.52	0.37	1.17 (1.08–1.27)	1.10 (1.01–1.19)	1.10 (1.02–1.20)	1.11 (1.02–1.20)
		*p*					<0.001	0.022	0.021	0.015
Age ≥ 85	High WC	No	61,021	48	297,969.31	0.16	1.00 (ref.)	1.00 (ref.)	1.00 (ref.)	1.00 (ref.)
		Yes	22,852	21	125,042.75	0.17	1.17 (0.70–1.95)	1.15 (0.69–1.92)	1.13 (0.68–1.88)	1.08 (0.65–1.81)
		*p*					0.548	0.598	0.650	0.767
	High TG	No	62,010	54	307,199.59	0.18	1.00 (ref.)	1.00 (ref.)	1.00 (ref.)	1.00 (ref.)
		Yes	21,863	15	115,812.47	0.13	0.79 (0.45–1.40)	0.77 (0.44–1.38)	0.78 (0.44–1.39)	0.76 (0.43–1.36)
		*p*					0.4136	0.3825	0.3937	0.3597
	High BP	No	35,852	24	170,861.12	0.14	1.00 (ref.)	1.00 (ref.)	1.00 (ref.)	1.00 (ref.)
		Yes	48,021	45	252,150.94	0.18	1.40 (0.85–2.30)	1.38 (0.84–2.26)	1.36 (0.83–2.24)	1.24 (0.75–2.03)
		*p*					0.185	0.211	0.224	0.406
	High Glucose	No	44,508	35	223,987.57	0.16	1.00 (ref.)	1.00 (ref.)	1.00 (ref.)	1.00 (ref.)
		Yes	39,365	34	199,024.49	0.17	1.10 (0.69–1.76)	1.09 (0.68–1.76)	1.08 (0.68–1.73)	1.12 (0.67–1.89)
		*p*					0.695	0.730	0.751	0.661
	Low HDL	No	46,931	41	246,144.58	0.17	1.00 (ref.)	1.00 (ref.)	1.00 (ref.)	1.00 (ref.)
		Yes	36,942	28	176,867.48	0.16	0.87 (0.54–1.41)	0.89 (0.54–1.48)	0.92 (0.55–1.53)	0.94 (0.56–1.57)
		*p*					0.568	0.656	0.737	0.808
